# Opportunities and Challenges of Understanding Community Assembly in Spontaneous Food Fermentation

**DOI:** 10.3390/foods12030673

**Published:** 2023-02-03

**Authors:** Maanasa Mudoor Sooresh, Benjamin P. Willing, Benjamin C. T. Bourrie

**Affiliations:** Department of Agricultural, Food, and Nutritional Science, Agriculture/Forestry Center, University of Alberta, Edmonton, AB T6G 2P5, Canada

**Keywords:** spontaneous fermentations, community assembly, microbial ecology, biotic selection, abiotic selection

## Abstract

Spontaneous fermentations that do not rely on backslopping or industrial starter cultures were especially important to the early development of society and are still practiced around the world today. While current literature on spontaneous fermentations is observational and descriptive, it is important to understand the underlying mechanism of microbial community assembly and how this correlates with changes observed in microbial succession, composition, interaction, and metabolite production. Spontaneous food and beverage fermentations are home to autochthonous bacteria and fungi that are naturally inoculated from raw materials, environment, and equipment. This review discusses the factors that play an important role in microbial community assembly, particularly focusing on commonly reported yeasts and bacteria isolated from spontaneously fermenting food and beverages, and how this affects the fermentation dynamics. A wide range of studies have been conducted in spontaneously fermented foods that highlight some of the mechanisms that are involved in microbial interactions, niche adaptation, and lifestyle of these microorganisms. Moreover, we will also highlight how controlled culture experiments provide greater insight into understanding microbial interactions, a modest attempt in decoding the complexity of spontaneous fermentations. Further research using specific in vitro microbial models to understand the role of core microbiota are needed to fill the knowledge gap that currently exists in understanding how the phenotypic and genotypic expression of these microorganisms aid in their successful adaptation and shape fermentation outcomes. Furthermore, there is still a vast opportunity to understand strain level implications on community assembly. Translating these findings will also help in improving other fermentation systems to help gain more control over the fermentation process and maintain consistent and superior product quality.

## 1. Introduction

Fermentation is among the oldest forms of food preservation, with potential evidence of fermentation dating back 13,000 years [1]. Fermented foods are defined as “Foods made through desired microbial growth and enzymatic conversions of food components” [2]. Our growing knowledge on the microbial diversity associated with food fermentations is continually being updated and recorded [3]. Fermented foods have gained their popularity not just for their excellent preservative aspects, but also for a number of health benefits they offer [4].

Food fermentations can proceed spontaneously, through backslopping, or by using a defined starter culture. Each of these methods select for microorganisms that vary in their lifestyle and adaptation [5] in addition to producing sensorially different end products [6]. Spontaneous food and beverage fermentations are characterised by the natural inoculation of microorganisms from raw materials, equipment, or environment [7,8]. These foods and beverages span across cereal, dairy, meat, vegetable and fruit-based substrates and include certain types of wine, beer, cheese, sauerkraut, miso, kimchi and cocoa, among many other traditionally produced spontaneous foods [6,9]. The raw materials and process conditions influence the composition of microorganisms associated with the fermentation [10]. These fermentation systems are quite dynamic with microbial succession over time being a common feature [11,12]. Commonly isolated microbial groups from spontaneous food fermentations include enterobacteria, lactic acid bacteria, acetic acid bacteria, yeasts, and moulds [13].

Although most industrial fermentations have proceeded to use defined starter cultures [14], this industrial trend, however, has left consumers craving fermented foods and beverages with unique flavour and aroma combinations [15]. For example, the craft beer industry is witnessing a re-emergence of spontaneously fermented speciality beers [16,17]. Craft breweries in the United States and other part of the world are adopting production processes similar to traditional Belgian speciality beers such as lambic and gueuze [18,19,20,21]. A similar trend is being observed in the wine industry, showing a shift from pure culture fermentations with *Saccharomyces cerevisiae* to spontaneous fermentations by autochthonous yeast and bacteria [22].

As the popularity of spontaneous fermentations soars, it becomes equally important to characterise and understand their fermentation dynamics to gain control over the process in order to achieve improved quality attributes [14]. In addition, spontaneous fermentations provide an opportunity to isolate strains that possess industrially relevant characteristics, including producing unique flavours [2,23]. Incorporating these strains in mixed fermentations can improve the flavour of the resulting products, in addition to gaining more control over the process [24].

Since spontaneous fermentations involve a complex consortium of microorganisms, the phenotypic and genotypic behaviour of an individual microorganism is very much dependent on the community assembly, which is strongly influenced by biotic and abiotic factors that govern the fermentation ecosystem [9]. The underlying mechanisms of microbial diversity, composition, and succession over time can be seen through the lens of community assembly, which is elucidated using the neutral and niche-based theory [25,26]. Neutral theory states that random or stochastic events are responsible for shaping community assembly, while niche-based theory expands on the idea that biotic and abiotic factors provide basis for the selection of microorganisms across space and time [27]. Combining the two theories, concepts in ecological community assembly can be explained by drift (random/stochastic events), diversification (genetic variations), dispersal (spatial movement) and selection (ecological fitness) [2,26,27,28].

While concepts in microbial ecology are still evolving [29] this review discusses community assembly in spontaneously fermented foods through the lens of biotic (lifestyle, domestication and microbial interactions based on genetic, phenotypic and metabolic traits), abiotic (temperature, salinity, pH, ethanol, oxygen, substrate) and other selective environmental factors (raw materials, equipment, environment, biogeography) that shape microbial diversity, succession, interaction, composition, and metabolite production, drawing examples from observational and experimental studies dissecting food fermentation systems. Understanding the implications of these factors in the selection of desirable characteristics in these food systems is crucial for their improved quality, safety and consistency [2].

## 2. Raw Materials

Microorganisms involved in spontaneous fermentations typically originate from raw materials, equipment, and the environment [30,31,32]. The ability of microorganisms to consume the initial nutrients available and their competitiveness to withstand the changing environmental conditions caused by active fermentation determines their fate throughout the fermentation [31,33,34].

Enterobacteria are commonly detected in the initial phases of several spontaneous fermentations including beer [18], mahewu [30], sauerkraut [35], cocoa bean [36], carrot juice [37] and kimchi [38] due to their association with the raw materials [5]. Enterobacteria in traditional spontaneous beer fermentations prevail during the first few days to weeks of fermentation and have been found to contribute to the production of 2,3-butanediol, acetic acid, lactic acid, succinic acid, ethyl acetate, and higher alcohols [39]. The underlying mechanism for their early presence is unclear, although they are present in malt during storage [32,40], and have been correlated with the availability of easily accessible carbohydrate substrates such as fructose, maltose, sucrose, and glucose roughly after 24 h of spontaneous fermentation initiation [33]. In industrial spontaneous beer production, lactic acid is added to the initial wort to prevent the growth of enterobacteria [19]. This is an example of how to naturally control spontaneous fermentations, although the influence of an enterobacterial phase on flavour formation is still poorly understood. These studies provide a strong foundation to speculate that occurrence of enterobacteria during the initial stages of spontaneous cereal fermentation may be a result of raw material association, although questions regarding their survival strategies and their influence on community assembly, functions, and flavour production as early members of spontaneous fermentations remain unanswered.

An interesting study was conducted to compare tomato juice fermentations that were started spontaneously, with an autochthonous and allochthonous strain of the same species of lactic acid bacteria [41]. The resulting products differed in cell densities of the selected strain, exopolysaccharide production, ascorbic acid, glutathione, total antioxidant activity, alcohols, aldehydes, ketones, sulfur compounds and esters. Specifically, tomato juice fermented with an autochthonous strain of *Lactiplantibacillus plantarum* POM1 and POM 35 showed higher cell densities and increased levels of ascorbic acid and glutathione compared to the spontaneous fermentation, and the fermentation initiated by an allochthonous strain of *Lactiplantibacillus plantarum* LP54 [41]. Cagno et al. reviewed fruit and vegetable fermentations whose performance in terms of acidification and sensorial characteristics was improved by the addition of autochthonous strains of lactic acid bacteria, highlighting their environmental adaptation benefits compared to allochthonous strains of lactic acid bacteria of the same species [42]. For spontaneous fermentations that are initiated with several types of raw material, such as kimchi, the initial microbial contribution of each raw material over the course of fermentation was studied; however, the microbial succession in the fermentations of individual raw materials did not resemble kimchi fermentations initiated with all the plant materials together [43]. Kimchi fermentations are characterised by the dominance of three groups—*Leuconotsoc*, *Weisella* and lactobacilli [44]. While Song et al., were able to map the dominant genera to cabbage and garlic, the underlying reasons behind the establishment of *Leuconostoc* in kimchi fermentations over a 50-day period is still unclear.

Identifying and tracking key microbial players during the first few days to weeks of spontaneous fermentations in multiple batches is important in understanding their role in the microbial succession that follows [18]. Comparing the microbiota of the raw materials and the microbiota associated with the course of fermentation will provide insight into how organisms are selected based on the changing fermentation conditions [45]. This can be seen in mahewu, a fermented cereal beverage from Zimbabwe. Through culture-dependent, RAPD-PCR and amplicon sequencing analyses, it was found that majority of mahewu’s microbiota closely matched the microbiota of the millet malt it was prepared from, although strain-specific qPCR showed that the most abundant strain present in the later stages of mahewu fermentation, *Lactiplantibacillus plantarum* FUA3590, was below detection in the starting material [30]. While these examples illustrate the role of raw materials in the establishment of the fermentation microbiota, the prevalence of specific strains or species of microorganisms not associated with raw materials at later stages of fermentations indicates that these microorganisms possess adaptive strategies, and their presence is not a result of random events.

## 3. Environment and Equipment

Bokulich et al. studied the pattern of microbial movement and distribution over time within a brewery environment that produces conventional, mixed, and spontaneous beer [32]. Raw materials such as barley, malt, hops, and the surfaces they were exposed to on a regular basis due to continuous production methods contributed to a higher microbial footprint when compared to other sources such as skin, air, and water [32]. These observations are in line with the concept of ‘dispersal’ being a key driver for community assembly in spontaneous fermentations [5]. Understanding factors that shape microbiota associated with an environment, such as raw materials, seasons, temperature, air flow, human handling, cleaning practices and building layout, may help in speculating the origin of a species, although insects and other vectors could also play a role in their dispersal [46,47]. In addition to microbial patterns associated with raw materials, different surfaces and sites within a processing environment as well as seasonal changes appear to influence the diversity and dispersal of microorganisms associated with wine and may be due to varying production processes, humidity and temperature fluctuations during production [32,48,49]. The effects of seasonal changes on the microbial composition of raw ingredients are yet to be analysed for other spontaneous fermentations and should be strongly considered as many spontaneous fermentation processes closely follow seasonal practices.

Yeasts and bacteria that become residents of production and processing sites adapt themselves to the environment and become a part of the fermentation ecosystem across space and time [50]. Evidence for this can be drawn from non-spontaneous fermentations, although the same concepts may still apply for industrial spontaneous fermentations. Bokulich and Mills found that processing facilities that produced cheeses inoculated with commercial starter cultures harboured microbes that were not part of the defined cultures. Of further interest is that individual species were correlated with different production sites associated with different processing steps [51]. This trend was observed across two different cheese facilities, showing that process parameters and their related surfaces retain specific microorganisms that adapt to the environment over time, in spite of cleaning procedures in place. These microorganisms were also detected in the maturing cheeses and high throughput sequencing data revealed that they were part of the dominant microbiota. A similar observation was noted in another study, where the fermentation of cheese inoculated with a yeast and bacterial mixed starter culture was, however, dominated by the microorganisms that were detected in the environment [52]. Microbial sampling of sake fermentation inoculated only with *Aspergillus flavus* var. oryzae showed a microbial succession of fungi and bacteria that were predominantly detected in the different sites of the production environment, with microorganisms from the family *Lactobacillaceae* being the most abundant among the environment and the fermentation microbiota [49]. These examples show that environmental microorganisms associated with spontaneous fermentations are driven by selection and become important members of the fermentation [2].

Within the context of understanding microbial distribution in a production environment, wooden barrels that are used for the maturation of wine and beer are said to be home to a number of microorganisms, which have been found to exist in a viable but non culturable (VBNC) state [17]. While there is no strict definition for spontaneously fermented beer as these beers are commonly defined as ’spontaneously’ inoculated, the use of wooden barrels from other fermentations such as wine may carry over some residual microbes, which could be seen as a mild form of backslopping. De Roos et al. sampled the interior surfaces of oak port wine casks used in the maturation of previously fermented beer [53]. The barrels were sampled before cleaning, after cleaning, after sulfuring and before filling the barrel with a fresh batch. Through amplicon sequencing the most abundant microbial species included *Brettanomyces* (teleomorph *Dekkera*), *Pichia*, *Acetobacter*, *Cellulosimicrobium*, and *Pediococcus*, although the cleaning stages sampled along with barrel age and type influenced their relative abundance [53]. *Brettanomyces bruxellensis and Pediococcus damnosus* have been reported to be associated with the maturation process of spontaneous beer fermentations, producing several important flavour compounds such as lactic acid, 4-vinyl guaiacol, 4-ethyl phenol, and esters [54]. *Brettanomyces bruxellensis* strains isolated from wine fermentations are also known to have developed tolerance against sulfite, a cleaning agent used in the wine industry: their ability to form biofilms may contribute to their ability to survive the harsh cleaning processes used on barrels [55]. These results indicate that microorganisms that persist in a food processing environment develop adaptive strategies that may enhance their functional role in the spontaneous fermentation they are a part of.

## 4. Microbial Biogeography

Microbial biogeography deals with the study of mechanisms underlying the abundance of microbial populations in a particular habitat [56]. Microbial biogeography can be used as a metric to understand the patterns of microbial dispersal, source limitation, dispersal limitation and establishment limitation from the field to the fermented product [57]. For example, Bokulich et al. utilised high throughput sequencing to identify factors influencing the microbial consortia inhabiting wine grape surfaces [56]. Interestingly, they found that the wine grape microbiota was associated with specific climactic features, with the growing region correlating to both fungal and bacterial composition in grape must regardless of grape variety or vintage. It is important to note, however, that analysis was not carried out on wine fermentations, so it is unclear if these differences impacted community assembly during fermentation. Concepts in source establishment limitation of lactic acid bacteria in cabbage phyllosphere was demonstrated by using a gnotobiotic model system using culture-based and amplicon sequencing. They also supplemented germ-free cabbage with LAB to determine if LAB-enriched cabbage produced better fermentation outcomes. When allowing the control (field grown) and LAB-enriched cabbage to ferment, the fermentation dynamics in both succeeded to produce sauerkraut, highlighting that the abundance of LAB in cabbage phyllosphere does not affect fermentation outcome significantly [57].

Although most of the microorganisms associated with spontaneous fermentations originate from raw materials, the microbial assembly in raw materials is a consequence of geographic locations [58], seasonal changes [48], climatic conditions, vectors, fields [56,59,60], climatic conditions [61] and phyllosphere microbiota [57]. Despite the raw materials harbouring several microorganisms, the microorganisms that are detected in fermentations seem to be environmentally adapted to the processing sites or fermentation environment [46], where they develop genetic strategies for their competitive advantages. Metagenomic tools can help identify the routes and sources of microbes associated with a processing environment [62]. The above-mentioned studies provide valuable guidelines for tracking microorganisms across space and time, especially for new fermentation facilities [46].

The following section explores the underlying genetic profiles of these autochthonous or ‘wild’ microorganisms.

## 5. Lifestyle and Domestication Events

It has been established that food fermentations are not the original habitats of lactobacilli, and they do not necessarily serve as a launchpad for speciation [50]. The lifestyle of fermentation-associated lactobacilli has been classified, based on a meta-phylogenetic data analysis, as free living (*Schleiferilactobacillus perolens*, *Latilactobacillus sakei*, *Paucilactobacillus vaccinostercus*, *Secundilactobacillus collinoides*, *Levilactobacillus brevis*, *Lentilactobacillus buchneri*)*,* host-adapted (*Ligilactobacillus ruminis*, *Limosilactobacillus reuteri*, *Lactobacillus amylovorus*, *Ligilactobacillus salivarius*, *Lactobacillus johnsonii*, *Lactobacillus iners*, *Lactobacillus apis*, *Bombilactobacillus mellis*, *Apilactobacillus kunkeei*) and nomadic (*Lactiplantibacillus plantarum*, *Lacticaseibacillus casei*, *Lacticaseibacillus rhamnosus*) [50]. A common trend that is observed is a larger genome size associated with free living and nomadic lactobacilli, and a short genome size in host-adapted lactobacilli, denoting their strict symbiotic lifestyle [50,63]. The genome size and the lifestyle of these bacteria are indicative of their carbohydrate substrate requirements. Information on the lifestyle of lactobacilli and other microorganisms associated with food fermentations provides a strong platform to infer the effects of dispersal tied with the genetic make-up of the microbe, providing insights into their adaptation to a fermentation ecosystem [50]. For instance, *Lactiplantibacillus plantarum* has often been noted for its nomadic lifestyle. Comparative genomics of 54 strains of *Lactiplantibacillus plantarum* isolated from different niches showed that this microorganism has a ‘flexible’ genetic makeup that supports survival in a wide range of environments as opposed to the gain or loss of strictly necessary genes that are needed to survive and thrive in a particular environment [64]. However, a correlation could not be drawn between its genome and its source of isolation. Martino et al. argue that adaptation to a specific environment may also be influenced by the expression of genes and not merely the presence or absence of genes [64]. A similar trend was observed in *Levilactobacillus brevis* brewery strains and insect-derived strains, where a conclusive correlation between their genomes and their isolation source could not be drawn [65]. These studies highlight the challenging nature of tracking a species or strain back to its original habitat, although conducting phylogenetic and comparative genomic analysis among different strains of the same species isolated from different spontaneous fermentations may serve as a useful tool to understand their mechanism of adaptation to a particular niche [66,67,68,69,70]. Horizontal gene transfer is another way by which microorganisms adapt to challenging environments [71]. Evidence of horizontal gene transfer in food fermentations has been reviewed [72]. Examples of likely horizontal gene transfer events in food fermentations include the presence of a plasmid encoded alpha-amylase gene in plant fermentation associated *Lactococcus lactis* IBB500, which was likely obtained from *Ralstonia* species or the presence of a unique *gal-lac* operon in fermented milk associated *Streptococcus infantarius* subsp. *infantarius* CJ18, which is homologous *Streptococcus thermophilus* [73,74]. There is also evidence of horizontal gene transfer in *Saccharomyces cerevisiae* strains [75], with a well-known example of a gene transferred potentially from *Saccharomyces pastorianus,* having functional implications in fructose transport system encoded by the FSY1 gene [76].

Domestication can be seen as a means of adaptation to an environment, in the presence of intentional or unintentional human intervention. Quoting Steensels et al., “Domestication is traditionally defined as the adaptation over time, especially by selective breeding, from a wild state of life in close association with and to the benefit of humans, causing morphological and physiological changes distinguishing domesticated taxa from their wild ancestors”. Domestication of microbes is an event in which continuous exposure to human influenced environments brings about selective pressures that push microorganisms to adapt to the new environment, in most cases, by losing or gaining genes [77]. In some cases, ‘wild type’ microorganisms can still survive and thrive in these harsh fermentation environments, without any changes to their genetic makeup [77].

While evidence of domestication events and diversification in fermentation related bacterial species are not conclusive [3], there is evidence of domestication in *Saccharomyces cerevisiae* strains in beer and wine recorded over the years [78]. Some of the adaptation mechanisms in ale and lager beer strains include the presence of an extra set of genes for maltose and maltotriose utilisation and the occurrence of flocculation [24]. Interspecific hybridisation has been a key factor in driving domestication among yeasts [79]. Examples from the food environment include *Saccharomyces pastorianus (Saccharomyces cerevisiae* × *Saccharomyces eubayanus*), *Saccharomyces bayanus (Saccharomyces cerevisiae* × *Saccharomyces uvarum* × *Saccharomyces eubayanus*), *Saccharomyces cerevisiae* × *Saccharomyces kudriavzevii*, *Saccharomyces uvarum* × *Saccharomyces eubayanus*, *Saccharomyces cerevisiae* × *Saccharomyces paradoxus*, and *Brettanomyces bruxellensis* [79]. Although there is no evidence yet in spontaneous fermentations, one can still argue that *Saccharomyces cerevisiae* yeasts associated with spontaneous fermentation, although referred to as ‘wild’ or ‘autochthonous’, have been, to an extent, indirectly domesticated by humans [80].

*Brettanomyces bruxellensis* strains isolated from wine and beer environments have been found to be genetically and phenotypically different from each other [67,81,82,83,84,85]. Specifically, the majority of wine isolates are found to be triploid, while strains isolated from beer environments are said to be haploid [81,86]. The triploid state of wine isolates has been hypothesised to have been a result of hybridisation events involving several species or sub-species of *Brettanomyces* to develop selective advantage of sulfite tolerance as the wine industry uses sulfur dioxide as a cleaning agent for their barrels [87].

Phylogenetic analysis conducted among naturally occurring hybrids of *Saccharomyces* isolated from different Belgian beer environments shows a divergence between ‘Trappist beer’ and ‘Lambic beer’ strains, which likely both originated from a single hybridisation event [88]. Analysis showed that ‘lambic strains’, which exist in a lower pH environment than Trappist strains, had higher tolerances to organic acids and low pH, potentially indicating niche adaptation in the lambic hybrid isolates [88].

## 6. Microbial Interactions

Patterns of community assembly of microbes is heavily influenced by microbial interactions, one such evidence being the microbial succession observed in spontaneous fermentations [89]. The outcomes of microbial interactions are defined in terms of mutualism, competition, commensalism, ammensalism and parasitism [90]. Positive interactions can occur in the form of physical contact, cross-feeding of metabolites, membrane contact, co-existence in biofilms and shared biomolecules such as enzymes [91]. Microbial interactions occurring in situ can often be challenging to decode because of the complexity of the microbial ecosystem dynamics [9]. For this reason, many studies have attempted to simulate spontaneous and mixed fermentation conditions with mock microbial communities to understand microbial interactions.

Studies that attempt to decode microbial interactions in food fermentations are designed by selecting different combinations of co-cultures of microorganisms that are the most abundant and that have been commonly isolated from the fermentation [92]. Microbial interactions of co-cultures can be tracked using growth experiments and compared against their growth in monoculture [89]. Interactions in co-cultures can also be followed by tracking metabolites produced by a particular microorganism and the subsequent consumption of the metabolites by another. There is evidence that microbial interactions play an important role in food fermentations and ultimately have an influence on product quality [93] and microbial community assembly [9]. A notable study by Cosetta et al. using a multispecies experimental community showed that volatile organic compounds (VOCs) produced by fungi can alter the microbial interactions and community assembly in cheese rind by driving the growth of *Vibrio* [94]; this observation was found to be true only at the genus level and not at the species or strain level. These observations led the authors to hypothesise that fungal VOCs may act as nutrient sources for particular bacterial genera in cheese rinds. While it may be too complex to map out each microbial interaction in a given food fermentation system, a simple model system can be used to study the microbial interactions between a few selected strains of interest. It is believed that the genes involved in microbial interactions are usually part of the core genome shared by microorganisms belonging to the same genus or family [89]. Nevertheless, given that microorganisms associated with food fermentations often acquire new and unique genes to adapt to the environment, it has been postulated that strain level diversity may influence microbial community assembly [95]. Niccum et al. used nine different strains of each bacterial species *Staphylococcus equorum*, *Brevibacterium auranticum* and *Brachibacterium alimentarium* isolated from nine different cheese rinds as their model communities. After inoculating the same cell count of each species in cheese curd agar, the impact of strain diversity was judged by measuring the colony forming units and the relative abundance of each species present in the communities. Although the nine model communities had the same species, they had different relative abundances of each species after 10 days. It is unclear whether the differences in community composition could be attributed to the strain level differences at a genetic level or at a phenotypic level influenced by the distinct environment they were isolated from [95]. There are a limited number of studies that focus on strain-level implications in a spontaneous food fermentation system. Inhibitory compounds such as bacteriocins and antibiotics produced by bacteria and fungi, respectively, have been identified in several food fermentations [96]. The production of bacteriocins and their mode of action have been reviewed previously [97,98]. While several bacteriocins such as weissellicin Y, weissellicin M [99], nicin, enterocin and pediocin [100] have been isolated and characterised from spontaneous fermentations, the role of these compounds in shaping community assembly in spontaneous fermentations is still unknown and requires further exploration.

The complexity of spontaneously occurring fermentations makes it challenging to map out all microbial interactions occurring during microbial succession. In addition, it also becomes challenging to judge which microbial interactions contribute to the important flavour compounds that are produced. Studies that employ synthetic communities of microorganisms for studying individual and coordinated interactions have provided insight into the interactions and underlying mechanisms that influence them. It is worth noting that the dominant taxa may not always be the ones that drive the overall composition and dynamics of a fermentation microbial ecosystem. Instead, ‘keystone taxa’, a term used to describe the taxa that influence the dynamics of a microbiota irrespective of their abundance, may also play a part in aspects of community assembly and fermentation dynamics including flavour production [101,102,103]. It becomes important to define the term ‘core microbiome’ of a fermentation not just in terms of the relative abundance of a particular taxon. The advent of inexpensive metatranscriptomic sequencing may help in the identification of possible keystone taxa, as this technique allows researchers to identify unique genes being expressed in a community that are essential for fermentation health. For example, Jung et al. found that, in kimchi fermentation, *Weissella koreensis* contributed approximately 35–65% of the relative expression levels of mRNA from day 18 to 29 despite accounting for only 20–25% of the microbial population as determined by amplicon sequencing [44]. Additionally, the expression levels of *Leuconostoc gelidum* were generally lower across this time period when compared to its relative abundance in the population. Additionally, metatranscriptomics has been used in cheese fermentations to examine the importance of non-starter lactic acid bacteria in the maturation process and showed that temperature significantly influenced the rate of proteolysis, lipolysis and amino acid/fatty acid catabolism [104].

It is interesting to note that a high relative abundance of a species may not solely correspond to the phenotypic and genotypic characteristics of the species but could be a result of its interaction with another species. Katsman et al. performed a study that compared three *Staphylococcus* species—*Staphylococcus xylosus*, *Staphylococcus equorum* and *Staphylococcus saprophyticus*—that are commonly encountered in cheese rinds. Among the three *Staphylococcus* species, *Staphylococcus equorum* had the highest relative abundance in the samples under evaluation. After performing pure culture and co-culture growth experiments with each of the three species, they noticed that *Staphylococcus equorum* was the slowest grower and was not a good competitor in the presence of other *Staphylococcus*. This led to the question of how *Staphylococcus equorum* had the highest relative abundance detected in shotgun metagenomic sequencing. After including other bacteria and fungi detected in the cheese rind microbiome, they were able to explain the high relative abundance of *Staphylococcus equorum* in cheese rind by its interaction with the fungus *Scopulariopsis*. This study highlights that relative abundance does not directly correlate with the microorganism’s standalone functionality. Specifically, it also shows how microbial interactions can influence microbial distribution. The study also showcases how co-culture experiments may not be enough to explain a complex fermentation system such as the cheese-rind ecosystem [102].

## 7. Abiotic Selection

Environmental factors such as pH, temperature, salinity, ethanol and moisture, depending on the fermentation system, play a key role in driving community diversity and succession through the different stages of fermentation [9]. These changes in the fermentation environment can result from both process parameters and the metabolism of microbes involved in different stages of fermentation [2]. Salt is one of the most common ways in which microbial composition is controlled in spontaneous fermentations ranging from olives to sauerkraut and is key in successful spontaneous vegetable fermentations. Yang et al. (2020) examined the impact of different salt concentrations on Chinese sauerkraut fermentations and found that a concentration of 2.5% was necessary to completely eliminate *Enterobacteriaceae* from the fermentation following 30 days of fermentation. They also found that a salt concentration of 2.5% resulted in the highest levels of lactic acid bacteria in the fermentation, while the 1.5% and 0.5% salt fermentations had higher levels of yeasts and *Enterobacteriaceae* and a 3.5% salt fermentation resulted in decreased lactic acid bacteria, yeast, and *Enterobacteriaceae*. Another study also found that salt concentrations over 2% resulted in lower levels of lactic acid bacteria, as well as lactic and acetic acid in sauerkraut [105]. Interestingly in radish paocai, lactic acid bacteria levels in the fermentation were negatively correlated with salt concentration, while yeasts increased under increasing levels of salt [106]. Enterobacterial growth in radish paocai was lower at day two of fermentation in the highest salt concentration of 7%, but this was the only difference observed. This study also conducted amplicon sequencing and found that among bacterial constituents, the relative abundance of the genus *Weissella* directly correlated to the salt concentration of the fermentation, while lactobacilli negatively correlated with salt concentration. *Pichia*, an important yeast in paocai, due to its ability to negatively impact final texture, was also impeded by 7% salt when compared to the other concentrations (0.01% vs. >2.5%). A similar effect of salt was found on both *Weissella* and lactobacilli in suancai, a fermented brassica product, as well as a positive correlation of *Pediococcus* with salt concentrations [107]. In fermented ground pork, it was found that increasing levels of salt resulted in increased catalase positive cocci populations and decreased enterobacterial populations over the course of fermentation, while lactic acid bacteria were unaffected by salt concentrations in the meat [108]. Isolates were also identified at the species level, with the species *Lactococcus lactis* observed at 0%, 1%, and 2% salt, but not 3% or 4%, indicating that species heterogeneity is an important factor in response to salt concentrations. The exact composition of salt used in fermentation can also impact community assembly in spontaneous fermentations. It has been shown that when 50% of the NaCl in spontaneous green table olive fermentation is replaced with other salts (KCl, MgCl, and CaCl_2_), the maximum *Enterobacteriaceae* population was approximately 4 log units higher than in the traditional process using 100% NaCl; however, by the end of fermentation, *Enterobacteriaceae* counts were significantly lower in all fermentations with approximately 1 log difference between traditional and non-traditional brine fermentations [109]. Additionally, it was found that traditional brine fermentation resulted in higher levels of yeasts throughout fermentation, while non-traditional brines resulted in 1.4–1.8 log_10_ increase in lactic acid bacteria on day 21 of fermentation with no differences observed at completion.

Temperature is another important component of the environment that influences the development of the fermentation microbiota. Indeed, temperature plays a critical role in daqu fermentation, with heat generated through microbial metabolism contributing to the temperature variation that is observed in three types of daqu fermentation—low (45–50 °C), medium (50–60 °C) and high temperature (60–65 °C). Due to the rapid growth of fungal taxa such as *Candida*, *Wickerhamomyces*, and many low abundance bacteria, the temperature of daqu increases to 53–56 °C resulting in a shift in microbial population to more thermotolerant genera including *Weissella*, *Bacillus*, *Thermoascus*, and *Thermomyces* in medium temperature daqu [110]. Additionally, levels of *Enterobacteriales* were shown to be higher in medium temperature daqu than in low temperature daqu at day 5 of fermentation, while *Lactobacillales* were higher in the low temperature fermentation at this time [111]. These findings may be due to the presence of locus of heat resistance genes in *Enterobacteriaceae* present in daqu fermentations allowing for better survivability in medium temperature fermentations [112], while in low temperature fermentations, lactic acid bacteria are better able to survive and produce lactic acid to drop the pH and limit *Enterobacteriaceae* growth (5 day pH of 5.28 vs. 4.87 in medium and low temperature daqu, respectively) [111]. Temperature is also an important factor influencing general community assembly, and species-specific dynamics of spontaneous meat fermentations. Utilising high throughput amplicon sequencing to examine spontaneous pork fermentation, van Reckem et al. (2021) and found that the bacterial population was altered by fermentation temperature, with temperatures of 23 °C and 30 °C resulting in fermentations dominated by *Leuconostoc and Lactobacillales*, while a 37 °C fermentation resulted in *Staphylococcus* becoming the dominant population alongside *Lactobacillales*. This study also found that the species-level diversity of staphylococci was altered by temperature as the dominant *Staphylococcus* was *Staphylococcus equorum* at 23 °C, *Staphylococcu epidermidis* at 30 °C, and *Staphylococcu aureus* at 37 °C. Interestingly, the authors indicated that there was little to no difference between fermentations with and without added glucose, indicating that temperature is a more important factor in the development of the fermentation microbiota and may act independently of carbohydrate availability [113]. These findings are similar to past work utilising culture-dependent techniques to examine how coagulase-negative staphylococci populations are impacted by fermentation temperature, which found that *Staphylococcus equorum* and *Staphylococcus aureus* dominated at 23 °C and 37 °C, respectively [114]. Stavropoulou et al. also found that *Latilactobacillus sakei* was the dominant species throughout fermentation at both 23 °C and 30 °C; however, upon increasing temperature to 37 °C it was found that *Pediococcus damnosus* dominated at 3 days and by day 14 there was an equal population of *Latilactobacillus sakei* and *Lactobacillus curvatus* [114].

The pH and titratable acidity of a fermentation environment can be altered through both process factors and the active metabolism of microorganisms present in the fermentation, and pH is possibly the most common factor influencing microbial composition during the fermentation process [115]. Indeed, in spontaneous wine fermentations it was demonstrated that the heterogeneity of *Saccharomyces* strains correlate with the total titratable acidity of the must, while sugar content had little impact on the dominant strains present in a fermentation [116]. In addition, the starting pH of a fermentation was found to significantly impact community assembly in ground pork fermentations, with decreased pH directly correlating to a nearly 3 log decrease in non-staphylococci in the fermentation following 14 days of fermentation [114]. It is important to consider that pH can change drastically during fermentation resulting in shifts in microbes. For this reason, it can be difficult to determine the specific impacts of a single factor in a fermentation system, as changes to common environmental conditions such as salt concentration or temperature can drastically alter the pH through time of a given fermentation. For instance, two studies found that as temperature increased in ground pork fermentation, so too did pH at both day 3 and 14 of fermentation [113,114]. These interactions have also been observed in daqu, with low temperature daqu fermentations exhibiting significantly lower pH levels at day 5 of fermentation than what was seen in medium temperature fermentations, possibly due to the increased level of lactic acid bacteria present in the low temperature daqu [111]. In both spontaneous vegetable and meat fermentations, salt concentration can directly impact pH, especially during the early stages of fermentation. Indeed, it has been shown in ground pork that pH decreased with increasing salt concentrations [108]. Similar interactions have been observed in vegetable fermentations [107,117,118]; however, some studies have observed the opposite interaction [105,106]. These different observations may be due to variation in the specific species and strains of organisms present in the raw materials used. These examples highlight the interconnected nature of the fermentation environment and the need to consider how altering one process factor may influence other important environmental pressures during the fermentation process. There is also a need to better understand how species or even strain level differences can impact these interactions, for instance with regards to the different results observed regarding salt concentrations and pH in vegetable fermentations.

While it has been shown that enterobacteria associated with daqu fermentations possess heat-resistant genes [112], their inability to withstand pH conditions below 4.5 in sourdough fermentations [119] leads to their eradication from the fermentation system, paving the way for microorganisms that are more acid tolerant. A recent study examining model carrot fermentations showed that the final cell count of enterobacteria was much lower in the fermentation with *Lactiplantibacillus plantarum* 299v when compared to its spontaneous fermentation counterpart, exhibiting that *Lactiplantibacillus plantarum* 299v can accelerate the inhibition of enterobacterial growth [120]. In addition, a drop in enterobacteria coincided with a more rapid drop in pH from day 3 in fermentations enriched with *Lactiplantibacillus plantarum* 299v, showing enterobacteria’s sensitivity to a pH of 3.5 in carrot fermentation [120]. The differences in pH tolerance between sourdough and carrot fermentation-associated enterobacteria indicate that organisms associated with different raw materials can have varying resistances to low pH. While inhibiting enterobacterial growth in fermentations may be desirable from a food safety perspective, functional implications of a suppressed enterobacterial count to a fermentation is unknown.

## 8. Conclusions

In conclusion, the microbial dynamics of spontaneous fermentations can be dissected systematically through concepts in community assembly. Particularly, we see that spontaneous fermentations are governed by ‘dispersal’ and ‘selection’ in every step of the process [2]. Dispersal is closely related to process conditions, wherever there is movement or exposure of substrate or fermenting material, along with the equipment and the environment. Metagenomic and amplicon sequencing can be used to trace patterns of microbial dispersal at the genus and sometimes species level across a food processing facility [32,46,62]. While selection also plays a key role during the initial steps of raw material processing, equipment, and environment exposure, it is also associated with the changing environmental conditions (pH, temperature, ethanol, moisture, salt concentration) within the fermentation ecosystem. Selection depends on genetic factors including microbial interactions, lifestyle of the microorganism, horizontal gene transfer and finally the expression of stress-related or adaptation-related genes. Tools such as comparative genomics, metatranscriptomics, and in vitro growth experiments have proven to be helpful in unfolding some of the underlying mechanisms in food fermentations [89]. While not all spontaneous fermentation studies have integrated the tools to evaluate these factors, it is necessary for future studies to use these factors as biomarkers to speculate that despite being spontaneous, the key microbes involved in these fermentations are adapted to the environment and become ‘reinoculated’ into the fermentations. In addition, there is also a need to understand how selection plays a role by looking at strain-level characterisation, as this will provide a platform to compare key yeasts and bacteria isolated from spontaneous fermentations from different batches, seasons, and geographical locations for conclusive evidence for the influence of strain level diversity on the flavour of the final product for maintaining consistency, to minimise fermentation failures and for best strain performance for use in mixed fermentation.

## Data Availability

No new data were created or analyzed in this study. Data sharing is not applicable to this article.
